# CircCEMIP promotes anoikis-resistance by enhancing protective autophagy in prostate cancer cells

**DOI:** 10.1186/s13046-022-02381-7

**Published:** 2022-06-02

**Authors:** Ying Yu, Yarong Song, Lulin Cheng, Liang Chen, Bing Liu, Dingheng Lu, Xuexiang Li, Yunxue Li, Fang Lv, Yifei Xing

**Affiliations:** 1 Department of Urology, Union Hospital, Tongji Medical College, Huazhong University of Science and Technology, Wuhan, 430022 China; 2 Present address: Department of Urology, Zhongnan Hospital, Wuhan University, 169 East Lake Road, Wuhan, 430061 China

**Keywords:** circ_0004585, TM9SF4, Anoikis, Autophagy, Prostate cancer

## Abstract

**Background:**

Circular RNAs (circRNAs) are essential participants in the development and progression of various malignant tumors. Previous studies have shown that cell migration-inducing protein (CEMIP) accelerates prostate cancer (PCa) anoikis resistance (AR) by activating autophagy. This study focused on the effect of circCEMIP on PCa metastasis.

**Methods:**

This study gradually revealed the role of circ_0004585 in PCa anoikis resistance via quantitative real-time PCR (qRT-PCR) analysis, western blotting, pull-down assays, and dual fluorescence reporter assays.

**Results:**

Functionally, circ_0004585 promoted PCa cells invasion and metastasis both in vitro and in vivo. Mechanistically, circ_0004585 directly interacted with miR-1248 to upregulate target gene expression. Furthermore, target prediction and dual-luciferase reporter assays identified transmembrane 9 superfamily member 4 (TM9SF4) as a potential miR-1248 target. Pathway analysis revealed that TM9SF4 activated autophagy to promote PCa cells anoikis resistance via mTOR phosphorylation.

**Conclusions:**

These results demonstrated that circ_0004585 played an oncogenic role during PCa invasion and metastasis by targeting the miR-1248/TM9SF4 axis while providing new insight into therapeutic strategy development for metastatic PCa.

**Supplementary Information:**

The online version contains supplementary material available at 10.1186/s13046-022-02381-7.

## Background

Anoikis is a form of programmed cell death, which is critically essential for the survival of human cancer cells after detachment from the extracellular matrix (ECM). Since aggressive anoikis-resistant cells are more likely to survive in the circulatory system, they are considered a vital prerequisite for cancer progression [1, 2]. However, the precise molecular mechanisms involved in PCa cells survival following ECM detachment remain unclear.

CEMIP, known as KIAA1199, was first described as an inner-ear-specific gene [3], which also played a role in developing and maintaining cancer metastasis. Recent clinical studies have shown that CEMIP increases aggressive tumor phenotypes by promoting hyaluronic acid depolymerization and the inclusion of membrane-associated clathrin in pit endocytosis [4, 5]. Furthermore, research has indicated that CEMIP is aberrantly expressed in multiple types of cancers, such as colorectal [6], gastric [7], breast [8], pancreatic [9], cervical [10], and liver cancer [11]. Recent studies by our team have shown that CEMIP is upregulated in PCa and promotes its progression by regulating metabolic reprogramming [12]. Furthermore, CEMIP promotes PCa cells anoikis resistance and metastasis by activating protective autophagy [13]. However, the regulatory and functional mechanisms of circCMEIP in tumorigenesis and aggression remain elusive.

Circular RNAs (circRNAs) are stable, covalently closed molecules resistant to exonuclide RNase R degradation [14]. Evidence shows that circRNAs are aberrantly expressed in gastric [15], colorectal [16], liver [17], PCa [18], and various other cancers and are essential for regulating the proliferation, migration, invasion, apoptosis, and therapeutic resistance of cancer cells [15, 19]. Moreover, circRNA acts as an endogenous sponge for miRNA to regulate target gene expression. For example, FUS-induced circRHOBTB3 facilitates cell proliferation via miR-600/NACC1-mediated autophagic response in pancreatic ductal adenocarcinoma [20]. In addition, circRNAs may bind and sequester RNA-binding proteins (RBPs) to regulate their functionality [21]. Research has indicated that the HNRNPL RNA binding protein facilitates circARHGAP35 formation, which is associated with poor survival rates of cancer patients [22]. Therefore, it is crucial to understand the circRNAs mechanism in cancer progression.

This study identifies one intronic circRNA generated from the CEMIP gene as a novel regulator of CEMIP-miRNA complexes and PCa progression. Circ_0004585 is upregulated in human PCa tissues and promotes the invasion, anoikis resistance, and metastasis of PCa cells. Mechanistically, this study reveals that circ_0004585 promotes TM9SF4 expression by binding with miR-1248 to activate autophagy, enhancing PCa cell invasion, anoikis resistance, and metastasis. Therefore, circ_0004585 may serve as an oncogene to promote PCa metastasis, while this study provides a novel therapeutic target for PCa.

## Materials and methods

### Patient tissue specimens and cell lines

A total of 60 sets of PCa tissue specimens and their corresponding normal prostate tissues were obtained from patients who underwent prostatectomy for PCa at the Department of Urology of the Union Hospital affiliated with Tongji Medical College between 2015 and 2019. Before sample collection, approval was obtained from the Institutional Review Board of the Tongji Medical College of the Huazhong University of Science and Technology. All specimens were classified according to the 2004 World Health Organization Consensus Classification and Staging System for prostate neoplasms. The anoikis-resistance model and cell culture were established using human androgen-independent PCa cell lines, PC-3 and DU145, which were obtained from the Shanghai Cell Bank, Chinese Academy of Sciences (Shanghai, China). Androgen-dependent C4–2 and LNCaP cell lines were donated by Prof. Xiaoping Zhang and Professor Jun Zhao (Union Hospital, Wuhan, China). The prostatic epithelial cell line RWPE-1 and androgen-dependent 22RV1 were obtained from the China Center for Type Culture Collection, CCTCC (Wuhan, China). The cells were maintained in RPMI 1640 medium (Hyclone, GE Healthcare Life Sciences, Logan, UT, USA) containing 10% fetal bovine serum (Biologic Industries, Kibbutz Beit Haemek, Israel) and 1% penicillin/streptomycin (Beyotime Institute of Biotechnology, Nanjing, China) at 37 °C in 5% CO_2_ and 95% humidity. The anoikis-resistance model was established by continuously culturing corresponding parental (P) cells in ultra-low-attachment 6-well plates (Corning, NY, USA) for 7 days, after which they were transferred to standard plates to allow adherence for 24 h. Re-adherent cells were deemed anoikis-resistance [12, 23, 24].

### RNA extraction, RNase R treatment, and PCR assays

The total RNA was isolated from the tissue and cell lines using a RNeasy Mini Kit (QIAGEN, Germany) according to the instructions of the manufacturer. RNase R treatment occurred at 37 °C with 3 U/mg of RNase R (Epicenter, WI, USA) for 15 min. Complementary DNA was synthesized using random primers and a PrimeScript RT Master Mix reverse transcription kit (Takara, Dalian, China) or a commercial miRNA reverse transcription PCR kit (Ribo-Bio, Guangzhou, China). Genomic DNA (gDNA) was isolated using a QIAamp DNA Mini Kit (QIAGEN, Germany), while quantitative real-time PCR (qRT-PCR) analysis was performed using an SYBR Premix Ex TaqTM kit (Takara). The differences between the circRNA and miRNA were normalized to GAPDH or U6 levels. All data were analyzed using the StepOnePlus Real-Time PCR System (Applied Biosystems, Carlsbad, CA, USA). Bulge-loop miRNA qPCR primers were obtained from RiboBio (Guangzhou, China). The primer details are listed in Supplementary Table 1.

### RNA-fluorescence in situ hybridization (FISH)

Cy3-labeled circ_0004585 and Dig-labeled locked nucleic acid miR-1248 probes were purchased from RiboBio (Guangzhou, China). The images were obtained using a fluorescent in situ hybridization kit (RiboBio) according to the instructions of the manufacturer. All data were analyzed using a Nikon A1Si laser scanning confocal microscope (Nikon Instruments Inc., Japan) and ModFit LT software.

### Pull-down assay with a biotinylated-A probe

The biotinylated-circ_0004585 probe was synthesized by RiboBio (Guangzhou, China). The sequence of the probe was just complemented to the back-spliced junction of circ_0004585 (listed in Supplementary Table 4). The pull-down assay with biotinylated miRNA was performed as previously described [25, 26] The RNA complexes combined on the beads were finally extracted using an RNeasy Mini Kit (QIAGEN, China) for further assessment. The pull-down assay with biotinylated miRNA miR-1248 mimics or mutants was synthesized by RiboBio (Guangzhou, China), while the bound RNAs were purified using a RNeasy Mini Kit (QIAGEN) for further analysis.

### Apoptosis detection

Non-transfected cell apoptosis was detected using FITC V Apoptosis and PE Annexin V Apoptosis Detection Kits (BD Biosciences, Franklin Lakes, NJ, USA). Briefly, cells (5 × 10^5^) were collected and incubated with FITC/propidium iodide (PI) for 15 min in the dark at room temperature, while the apoptosis index was determined using a flow cytometer (Beckman Coulter, Indianapolis, IN, USA). For the detachment-induced apoptosis assay, cells were incubated in ultra-low-attachment plates for 24 h before detection. PC-3 and DU145 cells were treated with 200 nM rapamycin (Rapa), or 10 mM 3-methyladenine (3-MA) (Selleck Chemicals, Houston, TX, USA) and 10 μM Chloroquine (CQ) (Selleck Chemicals, Houston, TX, USA) for 24 h, respectively, while isometric double-distilled water or DMSO was used as negative controls (NC). The reagents and drugs used in the study are detailed in Supplementary Table 2.

### Wound healing assay

PC-3 and DU145 cells were cultured in 6-well plates and scraped with the fine end of a 200 μl pipette tip (time 0 h). The cell migration was photographed 0 h and 24 h after injury using ten high-power fields. Remodeling was measured as the diminishing distance across the induced injury, normalized to the 0 h control, and expressed as relative migration.

### Transwell migration and Matrigel invasion assays

The cell migration was evaluated using a 24-well Transwell plate with 8.0-mm pore polycarbonate membrane inserts (Corning). Homogeneous single-cell suspensions (200 μl; 1 × 10^5^ cells/well) in serum-free mediums were added to the upper chambers, while 500 μl complete mediums were added to the lower chambers. After incubation for 24 h at 37 °C in a CO_2_ incubator, the migrated or invaded cells were fixed with ice-cold methanol and stained with 0.1% crystal violet for 15 min at room temperature. The migrated cells were counted in three randomly chosen fields using an inverted phase-contrast microscope (Olympus, Tokyo, Japan) at 100× magnification.

### Cell Counting Kit-8 (CCK-8) assay

The cell viability was determined using a CCK-8 assay (Supplementary Table 2). The PCa cells were starved in a medium containing 0.1% fetal bovine serum for 24 h. Next, approximately 2 × 10^3^ cells were evenly seeded into 96-well plates in triplicate. Then, CCK-8 solution (10 μl) was added to each well and incubated at 37 °C for 2.5 h. The OD value was measured at 450 nm after 1 d, 2 d, 3 d, 4 d, and 5 d, respectively, using a SpectraMax M5 microplate reader (MD, USA). The cell doubling times were calculated using GraphPad Prism 7.0 software (La Jolla, USA), while the OD values were used to perform the statistical analysis.

### Immunofluorescence staining assay

The mCherry-GFP co-labeled LC3BII/I adenovirus (Beyotime) was transferred to PCa cells in different treatment groups for 24 h [27]. Autophagy flux were detected in 2D culture, and a total of more than 20 cells under each condition were counted for quantification of autophagy and the images were amplified by 200 times. and the images were magnified 600 times. The nuclei were stained with DAPI, and the data were analyzed using a Nikon A1Si laser scanning confocal microscope (Nikon Instruments Inc., Japan).

### Transmission electron microscopy (TEM)

The cells were collected and fixed with 2.5% glutaraldehyde (Sigma, G7526) at 4 °C for 2 h. TEM of the autophagic and autolysosome ultrastructures in the cells was performed as described in a previous study [28]. The images were acquired using an 11-megapixel CCD camera (Olympus, Japan).

### Immunohistochemistry analysis

The primary antibody used to detect TM9SF4 was purchased from the Proteintech Group (Chicago, USA). The immunostaining images were captured using an Olympus FSX100 microscope (Olympus, Japan). The immunohistochemical expression level of TM9SF4 was evaluated by H-score, which was jointly completed by two pathologists in our hospital. The protein expression levels were analyzed by calculating the integrated optical density as described [29].

### Luciferase reporter assays

The TM9SF4 3′-UTR or promoter reporters were transiently transfected along with the Renilla control plasmid, while the PCa cells were co-transfected with miR-1248 mimics. The luciferase activities were measured for 24 h using a dual-luciferase reporter assay detection kit (Promega, WI, USA) as previously described [30].

### Plasmid construction and stable transfection

The human circ_0004585 and TM9SF4 3′-UTR cDNA was synthesized by TSINGKE (Wuhan, China). Circ_0004585 was cloned into a pCD-ciR vector (Geenseed Biotech Co, Guangzhou, China) containing front and rear circular frames. The TM9SF4 3′-UTR was cloned into a pMIR-REPORT vector. The T1 and T2 TM9SF4 3′-UTR plasmids and mutant luciferase reporters were synthesized using a Trelief™ SoSoo Cloning Kit (TSINGKE, Beijing, China). The TM9SF4 promoter-luciferase reporter vector was constructed and used as described in previous studies [31]. The miR-1248 mimics and the control were purchased from RiboBio (Guangzhou, China), while the siRNA aimed at circ_0004585 was synthesized by Gene-Pharma (Shanghai, China). The shRNA targeting TM9SF4 was designed and synthesized by Genechem (Shanghai, China). The targeted sequence was in Supplementary Table 5. while Lipofectamine 2000 (Life Technologies, USA) was used for plasmid transfection according to the instructions of the manufacturer. The transfected PCa cells were screened for four to six weeks using G418 (Life Technologies, USA).

### Western blots

The cell lysates were prepared with RIPA buffer (Thermo Scientific), the concentration of which was determined using a bicinchoninic acid protein assay kit (Pierce, Thermo Scientific). The immunoreactive bands were determined with an Immobilon ECL substrate kit (Millipore, Merck KGaA, Germany), while the images were acquired using a BioSpectrum 600 Imaging System (UVP, CA, USA). Detailed information regarding the primary antibodies used in this study is listed in Supplementary Table 3.

### Tumor xenografts

Four- to five-week-old male BALB/c nude mice were selected for the tumor xenograft experiments. PC-3 cells, labeled with Cy3 and transfected with overexpressed or knockdown circ_0004585 plasmids or control vectors, were subcutaneously injected into the tail veins of the mice (3 × 10^6^, 200 μl), who were sacrificed after six to seven weeks. No blinding occurred during the animal experiments. Xenograft images of the mice were obtained using the In-Vivo FX PRO (BRUKER Corporation, USA). All procedures were approved by the Animal Care Committee of Tongji Medical College.

### Statistical analysis

Statistical analysis of the data was performed using GraphPad Prism 7.0 software (La Jolla, USA), and the results were expresses as mean ± standard error of the mean (SEM). The two groups were compared via a two-tailed Student’s t-test. One-way analysis of variance was performed to evaluate the group differences, while *P* < 0.05 was considered statistically significant.

## Results

### Circ_0004585 is upregulated in the PCa tissues and cell lines and is mainly distributed in the cytoplasm

**Fig. 1:**
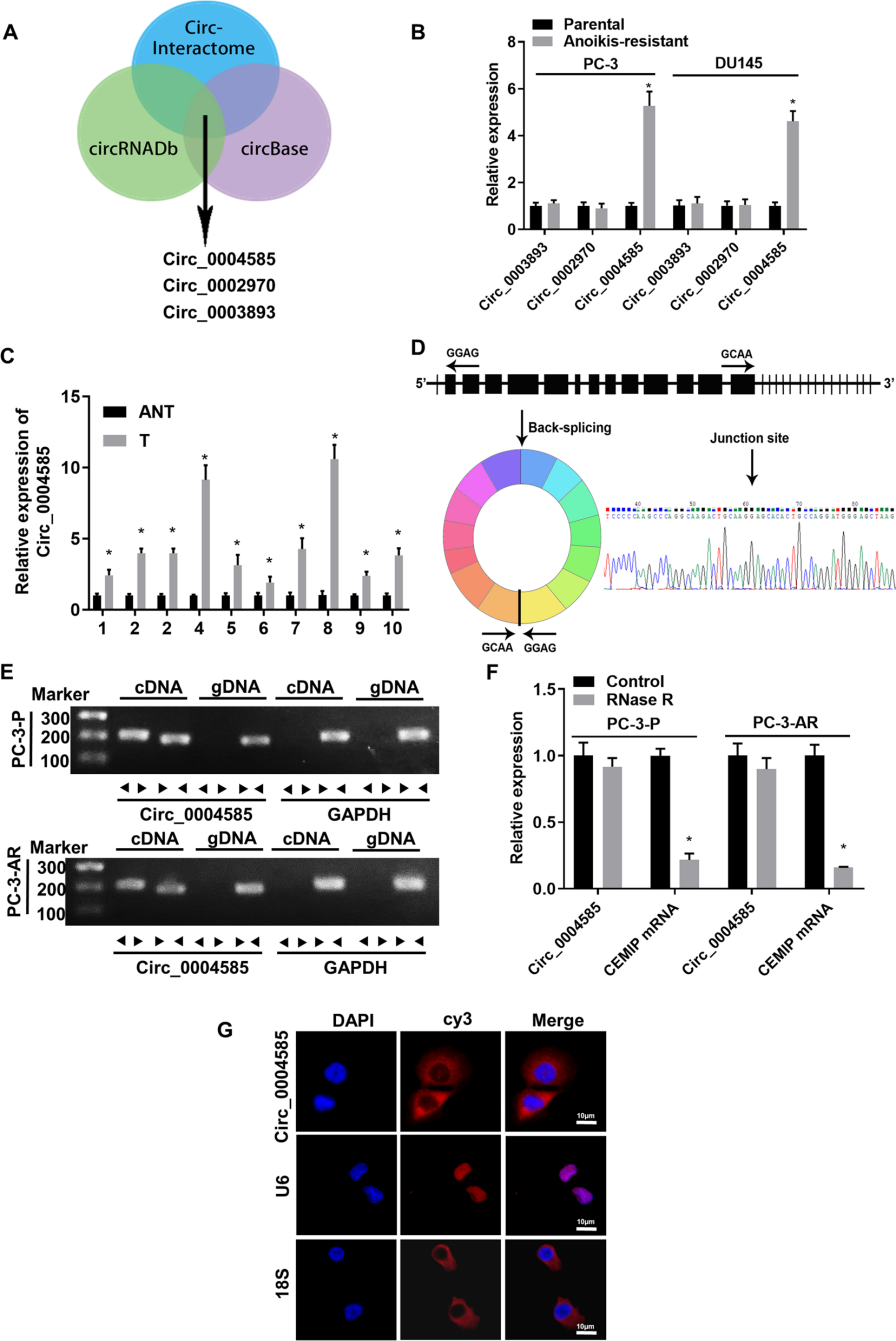
Circ_0004585 is upregulated in PCa tissues and cell lines, and mainly distributes in the cytoplasm. **A** The The Venn diagram screens out three CEMIP cyclization products. **B** qRT-PCR assay detect the expression level of circ_0004585 in anoikis-resistant PCa cells (PC-3-AR、DU145-AR) and the corresponding parent cells (PC-3-P、DU145-P). **C** qRT-PCR assay detect the expression level of circ_0004585 in 10 pairs of PCa and adjacent non-tumor tissue. **D** The schematic diagrams show that thirteen exons derived from CEMIP constitute circ_0004585. The existence of circ_0004585 was demonstrated by PCR and its back-splicing junction was verified by Sanger sequencing. **E** PCR assay with divergent or convergent primers indicating circ_0004585 is present in the PC-3 and DU145 cell lines. GAPDH was used as a negative control. **F** Real-time qPCR analysis of the expression of circ_0004585 after RNase R treatment in PC-3 and DU145 cells. **G** RNA-FISH indicates the location of circ_0004585. Nuclei were stained blue with DAPI. Circ_0004585 is stained red with Cy3 (Scale bar, 10 μm). Bar graphs show the statistical analysis of three independent experiments (* *P* < 0.05; ** *P* < 0.01)

First, the analysis of the circRNA sequencing databases, circInteractome, circBase, and circRNADb, showed that circ_0004585 was derived from the CEMIP gene (Fig. 1A). qPCR was used to measure the circ_0004585 expression in the parental and anoikis-resistant PCa cells, as well as ten PCa tissue samples and the corresponding adjacent normal tissue. The results showed that circ_0004585 expression was markedly upregulated in the anoikis-resistant PCa cells and PCa tissues compared with the parental PCa cells and adjacent normal tissue (Fig. 1B-C). Moreover, the circular CEMIP product was amplified with divergent primers and confirmed via Sanger sequencing (Fig. 1D). Next, the convergent and divergent primers were designed based on the cDNA and gDNA of the PC-3 and DU145 cell lines to amplify the linear and circRNA. Circ_0004585 was amplified by the divergent cDNA primers, while the gDNA yielded no amplification product (Fig. 1E). Real-time qPCR confirmed circ_0004585 resistance to RNase R, while the CEMIP mRNA was significantly reduced after RNase R treatment (Fig. 1F). RNA-FISH assays were then performed to identify the subcellular circ_0004585 localization using a specific Cy3-labeled probe. The images indicated that circ_0004585 was mainly localized in a punctate pattern in the cytoplasm (Fig. 1G). In addition, Supplementary Fig. 1 confirms that the ability of anoikis-resistance and migration in PCa-AR cells was significantly improved compared with PCa-P cells. These above results demonstrated that circ_0004585 was overexpressed in the PCa tissues and anoikis-resistant PCa cells and predominantly localized in the cell cytoplasm.

### Circ_0004585 promotes the autophagy and metastasis of PCa cells in vitro and in vivo

**Fig. 2:**
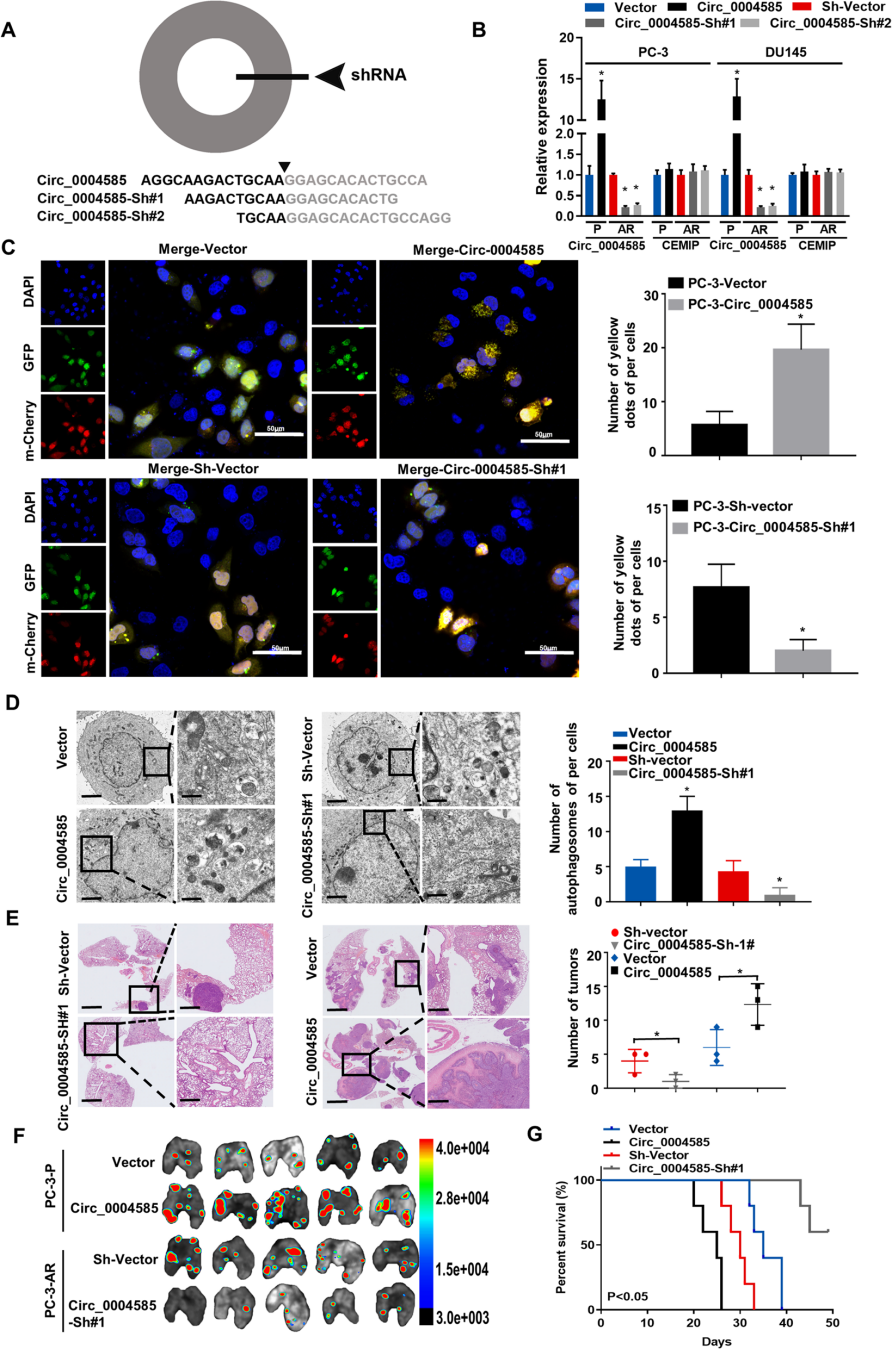
Circ000–4585 promotes autophagy and metastasis of PCa cells in vitro and in vivo. **A** Schematic representation of the sites of the siRNA specific to the back-splice junction of circ_0004585. **B** Real-time qPCR analysis of the expression of circ_0004585 in PC-3 and DU145 cells with stable overexpression or silencing. **C** Autophagic flux was monitored in stable overexpressed and downregulated circ_0004585 PC-3 cells expressing endogenous LC3BII/I tagged with tandem fluorescent-mCherry-GFP as a reporter. GFP and mCherry signal colocalization (yellow dots) indicated the lack of phagophore or autophagosome fusion with lysosomes (Scale bar,50 μm). **D** Transmission electron microscopy (TEM) revealed the number of double-membrane autophagosomes in stable overexpressed and downregulated circ_0004585 PC-3 cells (Original magnification, × 1000, × 1600, respectively). **E** HE stain assay showed that circ_0004585 knockdown compromised aggressive characteristics of circ_0004585 upregulated PC-3 cells, and inhibited in vivo pulmonary metastases (*n* = 5 per group, Original magnification, × 10, × 100, respectively). **F** Representative image of metastatic lung colonization in nude mice (n = 5 per group). **G** Kaplan–Meier curves for nude mice. Bar graphs show the statistical analysis of three independent experiments (n = 5 per group, * *P* < 0.05; ** *P* < 0.01)

Circ_0004585-overexpressing (circ_0004585) and circ_0004585-knockdown (circ_0004585-sh#1 and circ_0004585-sh#2) PC-3 and DU145 cell lines were established to investigate the impact of circ_0004585 on PCa cells migration, anoikis, and autophagy. The shRNA sequence is shown in Fig. 2A. Then, qRT-PCR was used to detect the circ_0004585 mRNA level in the PCa cells with stably overexpressed or downregulated circ_0004585 (Fig. 2B). In addition, the autophagic flux in the PCa cells expressing endogenous LC3BII/I tagged with tandem fluorescent-mCherry-GFP as a reporter was monitored to observe the autophagic changes in non-adherent conditions. GFP and mCherry signal colocalization (yellow dots) indicated the lack of phagophore or autophagosome fusion with lysosomes. As shown in Fig. 2C, more yellow puncta were evident after circ_0004585 overexpression in the PCa cells following detachment from the ECM for 24 h, while circ_0004585 downregulation reversed this effect. These results were further verified via TEM, revealing the presence of more double-membrane autophagosomes filled with degraded organelles and autolysosomes in the overexpressed circ_0004585 PCa cells (Fig. 2D). Then, the apoptosis level was assessed via flow cytometry to further determine the role of circ_0004585 during the anoikis process. The apoptosis rate of the downregulated circ_0004585 cells was significantly higher than the overexpressed circ_0004585 PCa cells in suspension conditions (Supplementary Fig. 2A). Moreover, wound healing assays showed that cells with significant circ_0004585 expression exhibited high mobility (Supplementary Fig. 2B). Similarly, the transwell assay indicated that circ_0004585 overexpression significantly enhanced the migration capacity of the PCa cells while reducing this trait in the circ_0004585-knockdown PCa cells (Supplementary Fig. 2C). To further confirm the role of circ_0004585 overexpression in PCa cells autophagic activation, anoikis resistance, and metastasis, the autophagic activator, rapamycin (Rapa), or the autophagic inhibitors, chloroquine (CQ) and 3-Methyladenine (3-MA), were added to PCa cells displaying stable circ_0004585 overexpression. Western blotting (Supplementary Fig. 2D), flow cytometry (Supplementary Fig. 2E), and transwell assays (Supplementary Fig. 2F) jointly and fully verified that circ_0004585 overexpression promoted the anoikis resistance and metastasis of PCa cells by activating protective autophagy. Furthermore, the autophagic activator, rapamycin, enhanced the biological effect, while autophagic inhibitors (CQ and 3-MA) achieved the opposite. To further investigate the impact of circ_0004585 on PCa cells metastasis in vivo, athymic nude mice, injected via the tail veins with PC-3 cells stably transfected with circ_0004585, displayed a higher number of metastatic pulmonary colonies, while their survival rate decreased (Fig. 2E-G). Therefore, all above the results confirmed that circ_0004585 overexpression promoted PCa cells anoikis resistance and metastasis by activating protective autophagy.

### Circ_0004585 directly interacts with miR-1248 in anoikis-resistant PCa cells

**Fig. 3:**
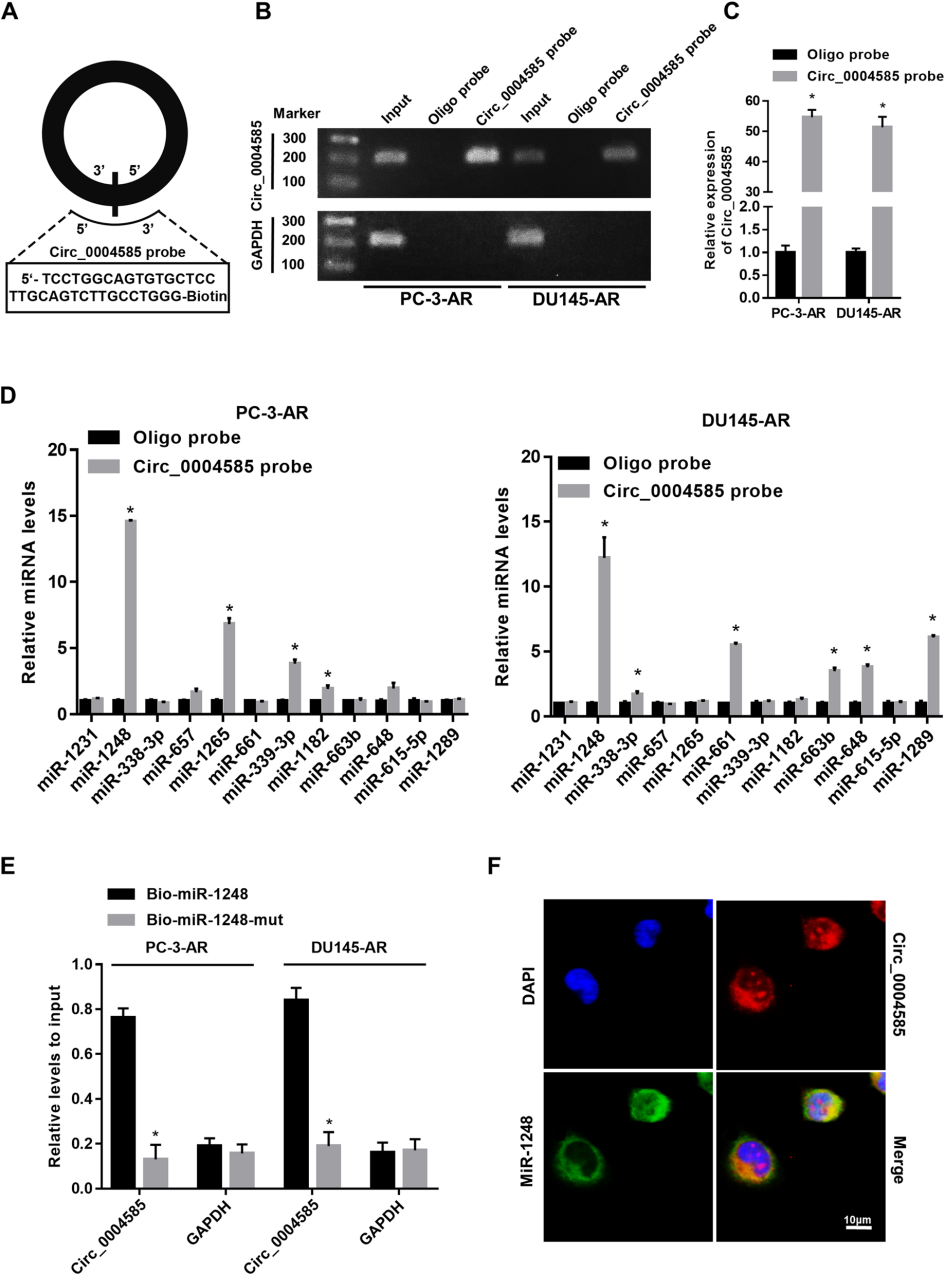
Circ_0004585 directly interacts with miR-1248 in anoikis -resistant PCa cells. **A** Schematic representation of the sites of the probe specific to the back-splice junction of circ_0004585. **B**, **C** Gel electrophoretic and qRT-PCR results showed that circ_0004585 could be specifically enriched by circ_0004585 probe. Relative level of circ_0004585 was normalized to input. GAPDH was used as negative control. **D** The relative expression levels of four miRNAs candidates were detected by qRT-PCR in PC-3 and DU145 cell lysates. **E** Circ_0004585 was enriched by biotinylated wild-type miR-1248 (Bio-1248-wt) or its mutant (Bio-1248-mut), and qRT-PCR was used to determine the relative circ_0004585 and GAPDH mRNA levels. **F** RNA FISH images showed the colocalization of circ_0004585 and miR-1248 in the cytoplasm of PC-3 cells (circ_0004585 probe was labeled with Cy3, locked nucleic acid miR-1248 was labeled with Dig, and the nuclei were stained with DAPI). Scale bar, 10 μm. Data are presented as the means ± SEM of three independent experiments (*P < 0.05; ** *P* < 0.01)

A circ_0004585 probe was constructed to clarify the propagative effect of circ_0004585 on PCa cells invasion and metastasis. Figure 3A presents a schematic representation of the probe sites specific to the back-splice junction of circ_0004585. The gel electrophoretic and qRT-PCR results provided in Fig. 3B, C showed that the circ_0004585 probe could specifically enrich circ_0004585. An ago2-RIP assay was conducted to search for genes binding to circ_0004585. The results showed that among the candidate miRNAs, miR-1248 was pulled down with the anti-ago2 antibody more effectively in the overexpressed circ_0004585 group than in the control (Fig. 3D). The qRT-PCR assay confirmed that circ_0004585 was enriched by biotinylated wild-type miR-1248 (Bio-1248-wt) rather than its mutant (Bio-1248-mut) in the PCa-AR cells, further verifying circ_0004585 and miR-1248 binding (Fig. 3E). The RNA-FISH images in Fig. 3F illustrated circ_0004585 and miR-1248 colocalization in the cytoplasm of the PC-3 cells (the circ_0004585 probe was labeled with Cy3, the locked nucleic acid miR-1248 was labeled with Dig, and the nuclei were stained with DAPI). These results suggest that circ_0004585 functions as a ceRNA for the miR-1248 in PCa cells.

### Circ_0004585 binds miR-1248 to upregulate the transcriptional activity of TM9SF4 and promote cell invasion in anoikis-resistant PCa cells

**Fig. 4:**
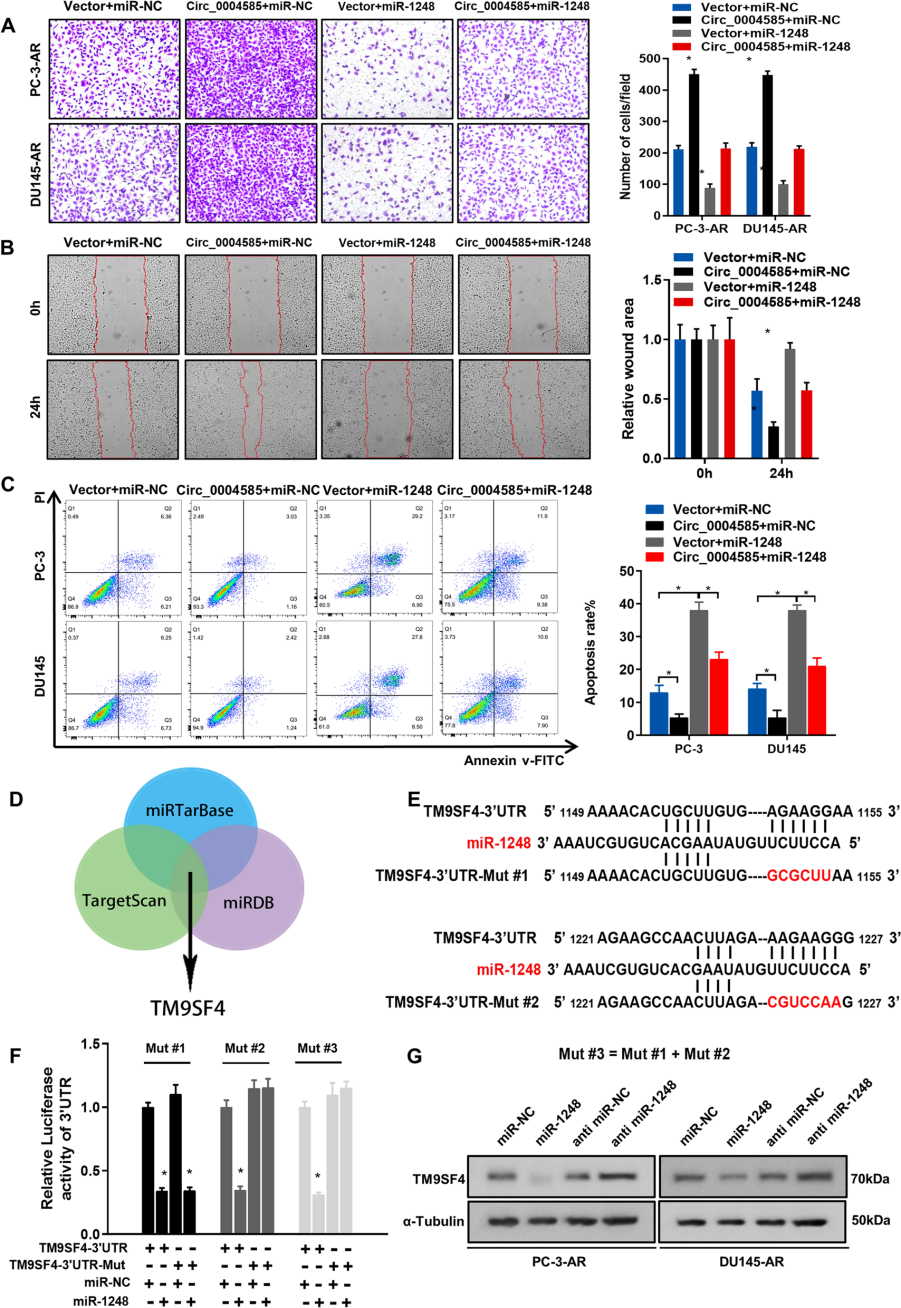
Circ_0004585 binds miR-1248 to upregulate the transcriptional activity of TM9SF4 and promote cell invasion in anoikis -resistant PCa cells. **A** Representative images (left panel) and quantification (right panel) results of transwell migration assay for PC-3 and DU145 transfected with miR-1248 mimics alone or cotransfected with circ_0004585 (Scale bar, 100 μm). **B** Representative images and quantification results of wound-healing assay for PC-3 and DU145 transfected with miR-1248 mimics alone or cotransfected with circ_0004585 (Scale bar, 100 μm). **C** Apoptosis was assessed by flow cytometry assay in PC-3 and DU145 transfected with miR-1248 mimics alone or cotransfected with circ_0004585. **D** The Venn diagram shows TM9SF4 is commonly predicted by TargetScan, miRDB and PicTarBase. **E** Schematic graph illustrated the mutation of potential binding site between miR-1248 and the 3′-UTR regions of TM9SF4. **F** Luciferase activity in PCa cells cotransfected with a luciferase reporter containing either TM9SF4 3′-UTR-wt or TM9SF4 3′-UTR-mut and miR-1248 mimics or inhibitors. **G** The protein levels of TM9SF4 detected by western-blotting in miR-1248 overexpressing and knockdown cells. Bar graphs show the statistical analysis of three independent experiments (* *P* < 0.05; ** *P* < 0.01)

The potential functional role of miR-1248 in PCa was assessed. The transwell migration and wound healing assays of PC-3 and DU145 transfected with miR-1248 mimics alone or cotransfected with circ_0004585 showed that enforced circ_0004585 expression significantly promoted PC-3 and DU145 cell migration, while miR-1248 mimic transfection had the opposite effect (Fig. 4A, B). To further confirm the effect of circ_0004585 binds miR-1248 on apoptosis of PCa, flow cytometry revealed that overexpression of circ_0004585 significantly promoted PCa cells anoikis-resistance. However, upregulated miR-1248 reversed this effect to a great extent (Fig. 4C). The autophagic-related TM9SF4 gene was predicted and determined using three databases (TargetScan, miRDB, and PicTarBase) to further examine the miR-1248 gene (Fig. 4D). The promoter regions and 3′-UTR-related luciferase reporters of TM9SF4 were constructed to provide further clarification regarding miR-1248-enhanced TM9SF4 expression. Figure 4E presents a schematic graph of the potential binding site mutants of miR-1248 and TM9SF4. Mut #1 and Mut #2 represented potentially mutated binding sites 1 and 2, while Mut #3 signified both potentially mutated binding regions. The results of a dual-luciferase reporter assay indicated a significant decline in the luciferase activity of the wild-type TM9SF4 3′-UTR reporter when the PCa cells were transfected with miR-1248 mimics. However, this effect was not observed in the mutated TM9SF4 3′-UTR, indicating that miR-1248 could bind directly to the TM9SF4 3′-UTR region, inhibiting its activity (Fig. 4F). Furthermore, western blot analysis showed that TM9SF4 expression was inhibited by miR-1248 upregulation and enhanced by miR-1248 inhibitor transfection (Fig. 4G). Therefore, the results revealed that circ_0004585 acted as a sponge for miR-1248 to upregulate the transcriptional activity of TM9SF4 and promote PCa cells invasion and anoikis-resistance.

### TM9SF4 overexpression is correlated with unfavorable pathological characteristics in PCa

**Fig. 5:**
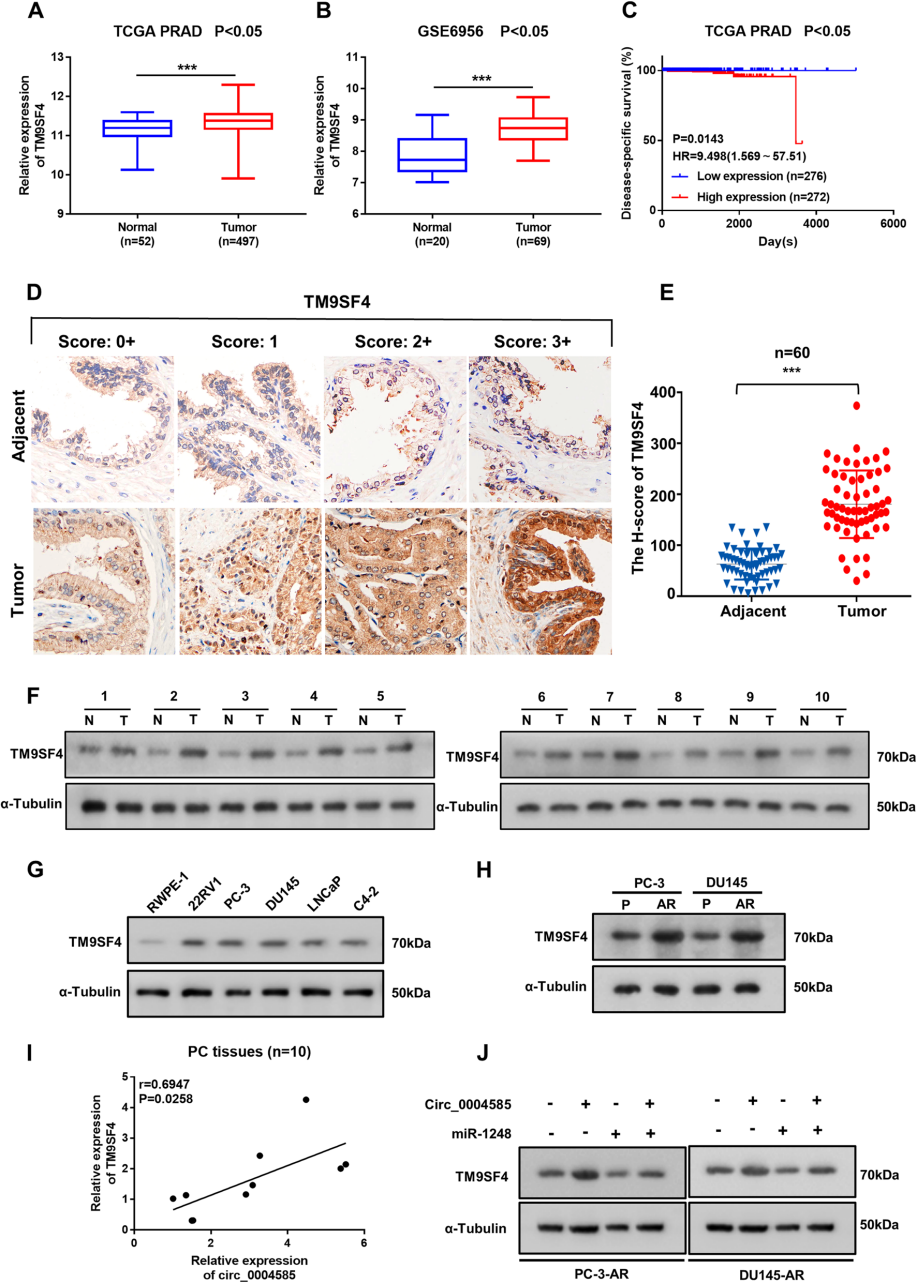
Overexpression of TM9SF4 is correlated with unfavourable pathological characteristics in PCa. **A**, **B** TM9SF4 expression in TCGA PRAD and GSE6956 dataset. **C** Disease-special survival in the TCGA PRAD dataset with low versus high levels of TM9SF4 mRNA, The cut-off value of TM9SF4 was used to distinguish between low expression (less than the cut-off value) and high expression (greater than or equal to the cut-off value). **D** TM9SF4 expression in 60 paired paraffin-embedded PCa and adjacent tissues detected by immunohistochemical staining (Original magnification, × 400). **E** Schematic representation of the H-score of TM9SF4 expression. **F**-**H** Western blotting assay showed the TM9SF4 protein expression in 10 pairs of PCa tissues, prostatic epithelial cells (RWPE-1), several PCa cell lines (22RV1, PC-3, DU145, LNCaP, C4–2), and anoikis-resisrant and corresponding parental PCa cells (PC-3, DU145). **I** Correlation of TM9SF4 and circ_0004585 expression in 10 pairs of PCa tissues. **J** The protein levels of TM9SF4 detected by western-blotting in circ_0004585 combined with miR-1248 overexpressing PC-3-AR and DU145-AR cells. Bar graphs show the statistical analysis of three independent experiments (* *P* < 0.05; ** *P* < 0.01)

Next, the TM9SF4 expression in PCa and its influence on PCa progression were explored. In this study of disease-specific survival curve, the cut-off value of TM9SF4 (11.36) was used to distinguish between low expression (less than the cut-off value) and high expression (greater than or equal to the cut-off value). The research databases, TCGA (https://www.cancer.gov/tcga) and GEO, GSE6956, https://www.ncbi.nlm.nih.gov/geo/query/acc.cgi?acc=GSE6956 jointly revealed significant TM9SF4 overexpression in PCa and were closely associated with the poor prognosis of PCa patients (Fig. 5A-C). The TM9SF4 expression was determined via the immunohistochemical staining of 60 paired paraffin-embedded PCa and adjacent tissues to further validate its pathological and clinical significance. Predominant TM9SF4 expression was evident in the cytoplasm, which was distinctly higher in the PCa tissues than in the normal paracancerous tissues (Fig. 5D, E). The TM9SF4 protein levels were significantly elevated in ten pairs of PCa tissues (Fig. 5F). The TM9SF4 expression in the prostate epithelial cells (RWPE-1) and several well-known metastatic cell lines (LNCaP, 22RV1, PC-3, and DU145) was determined via western blot analysis to explore the in vitro results. As expected, the metastatic PCa cells exhibited higher TM9SF4 protein levels than the RWPE-1 cells (Fig. 2G). What’s more, TM9SF4 is overexpression in PCa-AR cells compared with PCa-P cells (Fig. 2H). The correlation between the TM9SF4 expression and clinicopathological characteristics was analyzed, showing that TM9SF4 expression was positively correlated with the clinical PCa stage (I + II versus III + IV, *P* = 0.0364) (Table 1). Additionally, correlation analysis confirmed a significantly positive relationship between circ_0004585 and TM9SF4 (Fig. 5I). Moreover, although circ_0004585 expression substantially promoted TM9SF4 expression, a significant inhibitory effect was apparent in the presence of miR-1248 overexpression (Fig. 5J). In conclusion, these results suggested that the circ_0004585-regulated TM9SF4 expression in PCa is closely associated with poor prognosis.

**Table 1:** Clinicopathological characteristics of 60 cases of TM9SF4 expression by immunohistochemisty

Variable	n	TM9SF4		*P* value
		High(*n* = 47)	Low(*n* = 13)	
Age(y)				
<65(median)	26	19	7	0.0642
≥ 65	34	28	6	
PSA(ng/ml)				
<10	5	4	1	0.1744
10 ~ 20	21	16	5	
>20	34	27	7	
Gleason score				
≤ 6	6	3	3	0.1708
7	32	23	9	
≥ 8	22	21	1	
Pathological T Category				
pT1/2	30	20	10	0.0637
pT3/4	30	26	4	
Lymph node metastasis				
N0	50	39	11	0.4027
N1	10	8	2	
Stage classification				
I-II	24	23	1	0.0364*
III-IV	36	33	3	

### TM9SF4 activates autophagy by inhibiting mTOR phosphorylation to promote PCa cell anoikis-resistance and metastasis

**Fig. 6:**
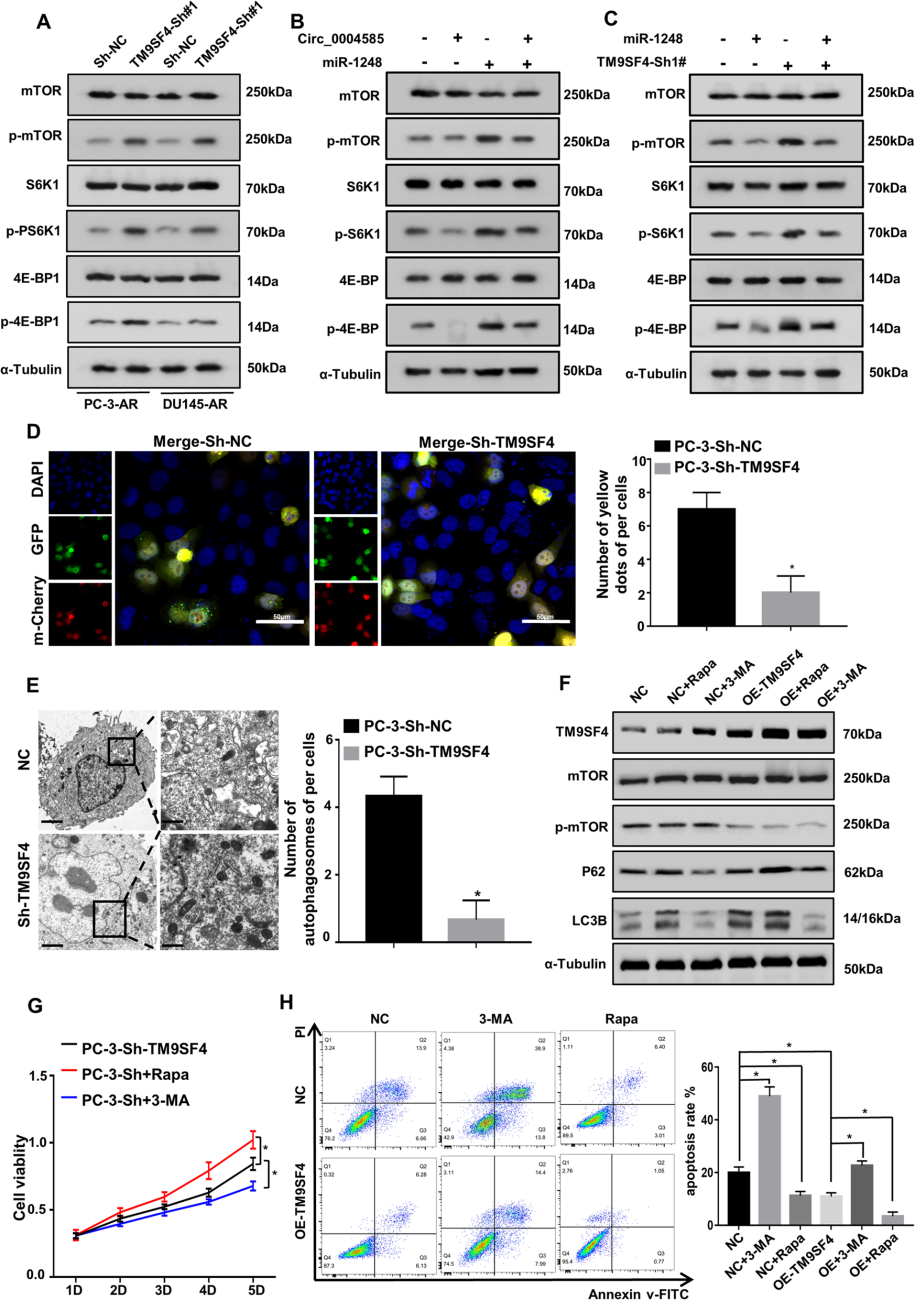
TM9SF4 activates autophagy via inhibiting mTOR phosphorylation to promote PCa cells anoikis-resistance and metastasis. **A** The protein levels of mTOR pathway proteins (mTOR, p-mTOR, S6K1, p-S6K1, 4E-BP1, and p-4E-BP1) detected by western-blotting in TM9SF4 silenced PC-3 cells. **B** Western-blotting showed that circ_0004585 and miR-1248 co-regulate the protein level of mTOR, p-mTOR, pS6, p-pS6, 4E-BP1, and p-4E-BP1. **C** The protein levels of mTOR, p-mTOR, pS6, p-pS6, 4E-BP1, and p-4E-BP1 detected by western-blotting in PC-3 cells transfected with knocking down TM9SF4 alone or cotransfected with miR-1248. **D** Autophagic flux was monitored in stable down-regulated TM9SF4 PC-3 cells expressing endogenous LC3BII/I tagged with tandem fluorescent-mCherry-GFP as a reporter. GFP and mCherry signal colocalization (yellow dots) indicated the lack of phagophore or autophagosome fusion with lysosomes (scale bar,50 μm). **E** Transmission electron microscopy (TEM) revealed the number of double-membrane autophagosomes in stable down-regulated TM9SF4 PC-3 cells. **F** Western blot assay detected the expression levels of TM9SF4 and autophagy related proteins (mTOR, p-mTOR, P62, and LC3BII/I) after the addition of Rapa or 3-MA for 24 h. **G** The cell viability rate was detected using CCK-8 after individually adding Rapa or 3-MA to the PCa cells displaying stable TM9SF4 knockdown. **H** Flow cytometry was used to detect the apoptosis of PC-3 cells with stable overexpression of TM9SF4 after exogenous addition of Rapa or 3-MA for 24 h. Bar graphs show the statistical analysis of three independent experiments (* *P* < 0.05; ** *P* < 0.01)

Stable overexpressed and knockdown TM9SF4 PCa cells were constructed to further examine the mechanisms underlying anoikis resistance and autophagic TM9SF4 regulation. First, qRT-PCR was used to detect the PCa cell mRNA levels after TM9SF4 overexpression and knockdown (Supplementary Fig. 3A). Next, the western blot assay revealed that TM9SF4 overexpression activated autophagy by inhibiting mTOR (Supplementary Fig. 3B). However, TM9SF4 knockdown facilitated the significant activation of the mTOR pathway by promoting mTOR phosphorylation to inhibit autophagy (Fig. 6A). As shown in Fig. 6B, the overexpressed circ_0004585 and miR-1248 plasmids were cotransfected to confirm the role of circ_0004585 in the mTOR pathway. The overexpression of circ_0004585 significantly inhibited the mTOR pathway, a phenomenon that was reversed by miR-1248 overexpression. In addition, the TM9SF4 knockdown and overexpressed miR-1248 plasmids in the PCa cells were subjected to further co-transfection, revealing that the upregulated miR-1248 inhibition of the mTOR pathway was significantly reversed by TM9SF4 knockdown (Fig. 6C). The autophagic flux was monitored in the PC-3 cells exhibiting TM9SF4 overexpression and downregulation to examine the autophagic changes in non-adherent conditions. As shown in Fig. 6D and Supplementary Fig. 3C, the autophagic flux level of the overexpressed TM9SF4 PC-3 cells increased significantly while declining substantially in the TM9SF4 knockdown PC-3 cells. Furthermore, the number of autophagosomes observed via TEM displayed the same effect in Fig. 6E and Supplementary Fig. 3D. Moreover, the autophagic activator, rapamycin, significantly increased TM9SF4-activated autophagy (P62, LC3BII/I), while this process was substantially restricted by the autophagic inhibitor, 3-MA (Fig. 6F). Next, the effect of the autophagic level on PCa cells anoikis was investigated. The cell viability rate was determined using CCK-8 after individually adding rapamycin or 3-MA to the PCa cells displaying stable TM9SF4 overexpression and knockdown. The rapamycin promoted PCa cells anoikis resistance, while a contradictory effect was achieved by 3-MA (Fig. 6F and Supplementary Fig. 3E). Moreover, flow cytometry demonstrated that rapamycin activated autophagy to reduce apoptosis in the upregulated TM9SF4 PC-3 cells, while 3-MA significantly promoted apoptosis by inhibiting autophagy (Fig. 6G and Supplementary Fig. 3F). In addition, the transwell assay confirmed that TM9SF4 overexpression promoted PCa cells migration (Supplementary Fig. 3G). Therefore, these results confirmed that upregulated TM9SF4 promoted PCa cells anoikis-resistance and metastasis by inhibiting mTOR phosphorylation to facilitate autophagic activation.

## Discussion

CircRNAs are a class of endogenous non-coding RNAs characterized by their covalently closed-loop structures [14], the abundance and diversity of which in mammalian cells have been confirmed by recent studies [32]. Hundreds of circRNAs reportedly function as vital tumorigenic drivers or tumor suppressors in human cancers [33]. For example, circPLCE1 is downregulated in colorectal carcinoma tissues and is correlated with advanced clinical stages and poor survival [34]. However, significantly higher circURI1 expression is evident in gastric cancer than in the corresponding adjacent nontumor specimens, inhibiting cell migration and invasion in vitro and gastric cancer metastasis in vivo [35]. Previous studies by our team have shown that CEMIP is upregulated in PCa and promotes PCa cell anoikis-resistance via autophagic activation [13]. This study further explored the role of CEMIP and circCEMIP (hsa_circ_0004585) cyclization products in PCa progression, revealing that circCEMIP was significantly upregulated in the PCa tissues and cell lines. Both in vivo and in vitro experiments indicated that circ_0004585 promoted PCa cell anoikis-resistance and metastasis, which could play an important oncogenic role in PCa while showing promise as a novel target for future PCa therapy. However, compared with linear mRNA, circRNA is more stable and easier to detect in cells, plasma, and even circulating exosomes [36–38]. Therefore, although further validation is required, circ_0004585 can possibly be used for the PCa diagnosis and prognosis evaluation.

Studies have suggested that circRNAs act as miRNA sponges and display the following characteristics: First, they are derived from one or more exons of known protein-coding genes via back-splicing [38]. Second, these circRNAs are predominantly found in the cytoplasm, similar to miRNAs [39, 40]. Finally, circRNAs with more predictive putative miRNA binding sites are likely to act as ceRNA for miRNA [41]. This study has shown that circ_0004585, derived from 13 CEMIP gene exons and primarily located in the cytoplasm, can combine with miR-1248 to regulate downstream target genes and promote protective autophagy in PCa cells. Furthermore, circ_0004585 overexpression significantly promotes the lung metastasis of PCa in vivo, while circ_0004585 downregulation significantly reverses this effect. However, whether circ_0004585 is involved in other biological processes, such as RNA protein binding [42–44] or peptide translation [45, 46], requires further confirmation.

Aberrant miRNA expression in various types of human cancer is pivotal during tumorigenesis, progression, and metastasis [47, 48]. Although miR-1248 is reportedly closely associated with the progression and prognosis of multiple types of cancer, including lung cancer [49, 50] and PCa [51], its specific role in PCa remains unclear. This study preliminarily confirmed that circ_0004585 promoted PCa cell autophagy and anoikis-resistance via miR-1248 sponging and binding, while miR-1248 overexpression reversed this biological effect. Therefore, this study revealed that miR-1248 inhibited PCa progression and metastasis, presenting a potential therapeutic target, but its specific regulatory mechanism requires further investigation.

TM9SF4, also known as TM9SF or nonaspanins, belongs to the transmembrane 9 protein family [52] and participates in cell adhesion, phagocytosis, and autophagy [53–55]. TM9SF4 is reportedly involved in the cannibalistic activity of metastatic melanoma cells and the resistance of colon cancer cells to chemotherapy by promoting the acidification of the tumor environment on the interior and exterior of the cell [56, 57]. However, no research is available regarding the biological functions of TM9SF4 in PCa. This study confirmed the significant overexpression of TM9SF4 in PCa, which was closely related to its clinical grade and poor prognosis. Moreover, TM9SF4 overexpression promoted PCa cell anoikis-resistance by inhibiting mTOR activity to facilitate autophagy, while the miR-1248 transcriptional regulation of TM9SF4 significantly promoted this effect. The inactivation of the mTOR pathway was essential for autophagic activation, providing energy support for PCa cell anoikis resistance and metastasis [13]. Furthermore, circ_0004585 encouraged PCa progression via miR-1248 sponging, promoting TM9SF4 expression. The comprehensive in vitro and in vivo experiments in this study revealed that circ_0004585 promoted TM9SF4 transcription via miR-1248 sponging, inhibiting mTOR phosphorylation to facilitate protective autophagy and anoikis-resistance in PCa cells. However, many aspects in this study remain unclear and require further exploration. For example, future research should clarify whether additional circCEMIP regulatory mechanisms are involved in PCa progression and find a balance between the protective and damaging impact of autophagy in PCa. Other issues requiring attention involve the exact regulation of mTOR phosphorylation and activity by TM9SF4 and whether additional TM9SF4 regulatory mechanisms are involved in PCa progression. The clarification of these issues denotes the objectives of our future research.

## Conclusions

In conclusion, this study reveals that the novel circRNA, circ_0004585, is upregulated in PCa tissues and cell lines, promoting PCa cell anoikis-resistance and metastasis both in vitro and in vivo. Moreover, circ_0004585 upregulated TM9SF4 to inhibit mTOR activity by binding miR-1248 to activate autophagy. Therefore, the results clarify the potential circRNA-related mechanisms behind PCa cell anoikis-resistance regulation while highlighting circ_0004585 as a promising therapeutic target for PCa treatment.

## Supplementary Information

**Additional file 1: Supplementary Figure 1.** Anoikis-resistant PCa cells enhance the capabilities of survival and migration. **Supplementary Figure 2.** Circ_0004585 promotes PCa cells migration, invasion, anikis-resistance. **Supplementary Figure 3.** Upregulation of TM9SF4 promotes the invasion, migration and anoikis-resistance of prostate cancer cells by activating autophagy. **Supplementary Table 1.** qRT-PCR primer sequences in this study. **Supplementary Table 2.** Drugs and reagents. **Supplementary Table 3.** Primary and secondary antibodies. **Supplementary Table 4.** Probe sequence in this study. **Supplementary Table 5.** TM9SF4 shRNA sequence in this study.

## Data Availability

The datasets used and/or analysed during the current study are available from the corresponding author on reasonable request.

## Abbreviations

circRNAs: Circular RNAs
PCa: Prostate cancer
AR: Anoikis resistance
circ_0004585: hsa-circ_0004585
qRT-PCR: Quantitative real-time PCR
TM9SF4: Transmembrane 9 superfamily member 4
ECM: Extracellular matrix
RBPs: RNA-binding proteins
TEM: Transmission electron microscopy
