# Supplementary material for: CircCEMIP promotes anoikis-resistance by enhancing protective autophagy in prostate cancer cells
Source: J Exp Clin Cancer Res. 2022 Jun 2;41:188. doi: 10.1186/s13046-022-02381-7 (PMC9161511; doi:10.1186/s13046-022-02381-7)
Supplement: Supplementary file 1 — Additional file 1: Supplementary Figure 1. Anoikis-resistant PCa cells enhance the capabilities of survival and migration. Supplementary Figure 2. Circ_0004585 promotes PCa cells migration, invasion, anikis-resistance. Supplementary Figure 3. Upregulation of TM9SF4 promotes the invasion, migration and anoikis-resistance of prostate cancer cells by activating autophagy. Supplementary Table 1. qRT-PCR primer sequences in this study. Supplementary Table 2. Drugs and reagents. Supplementary Table 3. Primary and secondary antibodies. Supplementary Table 4. Probe sequence in this study. Supplementary Table 5. TM9SF4 shRNA sequence in this study. [file 13046_2022_2381_MOESM1_ESM.pdf]

Supplementary Figure 1

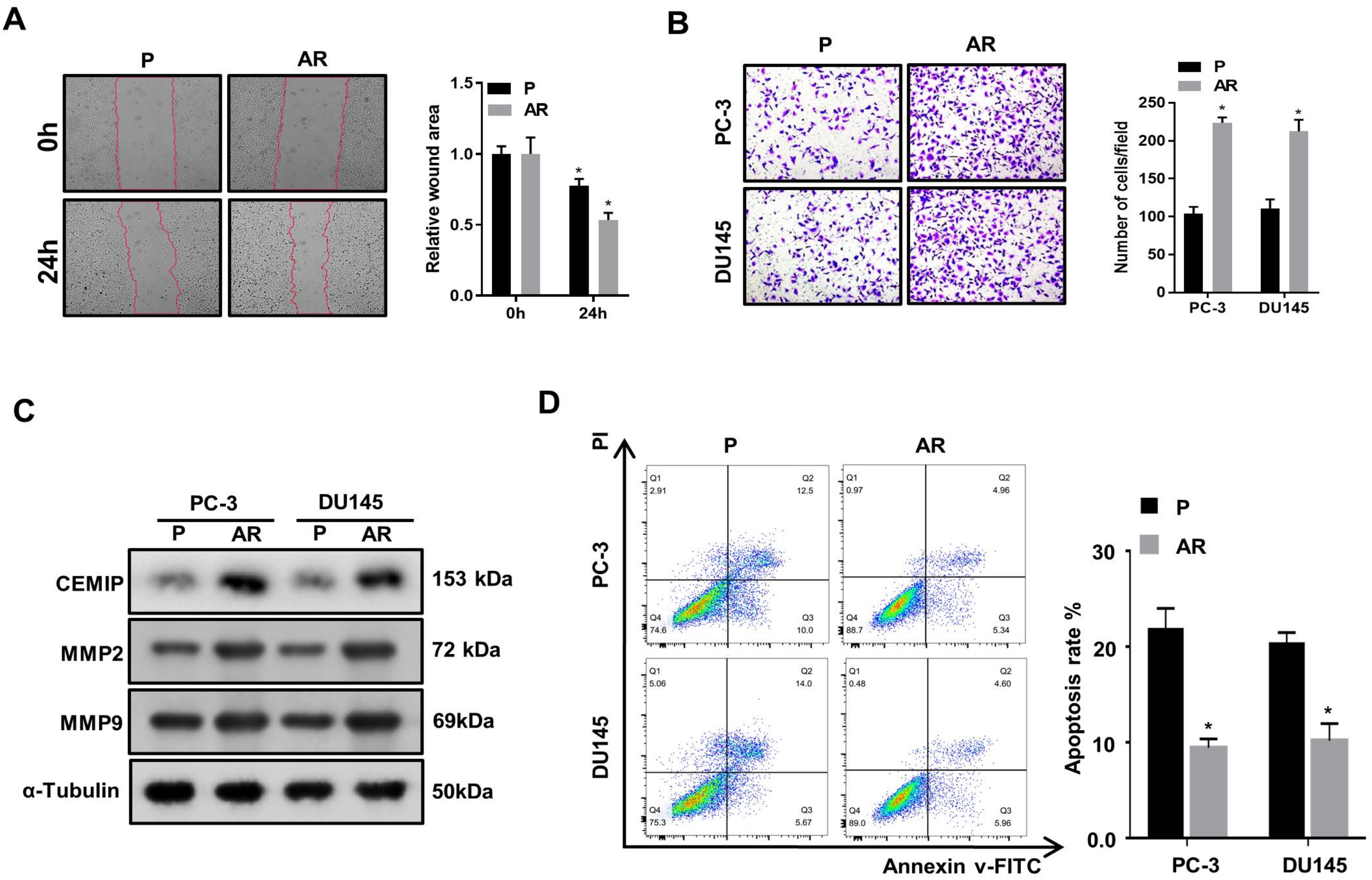

Supplementary Figure 2

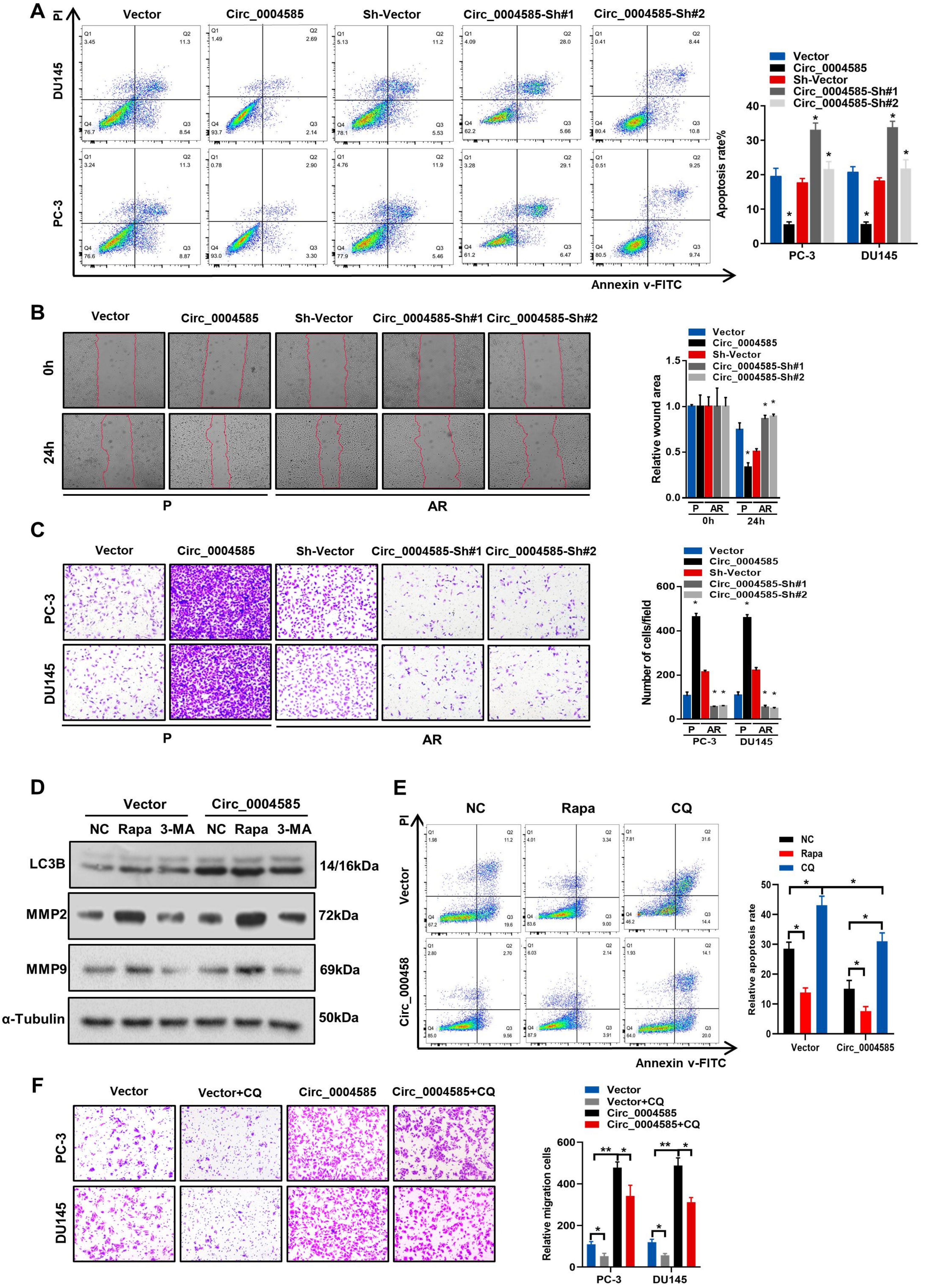

Supplementary Figure 3

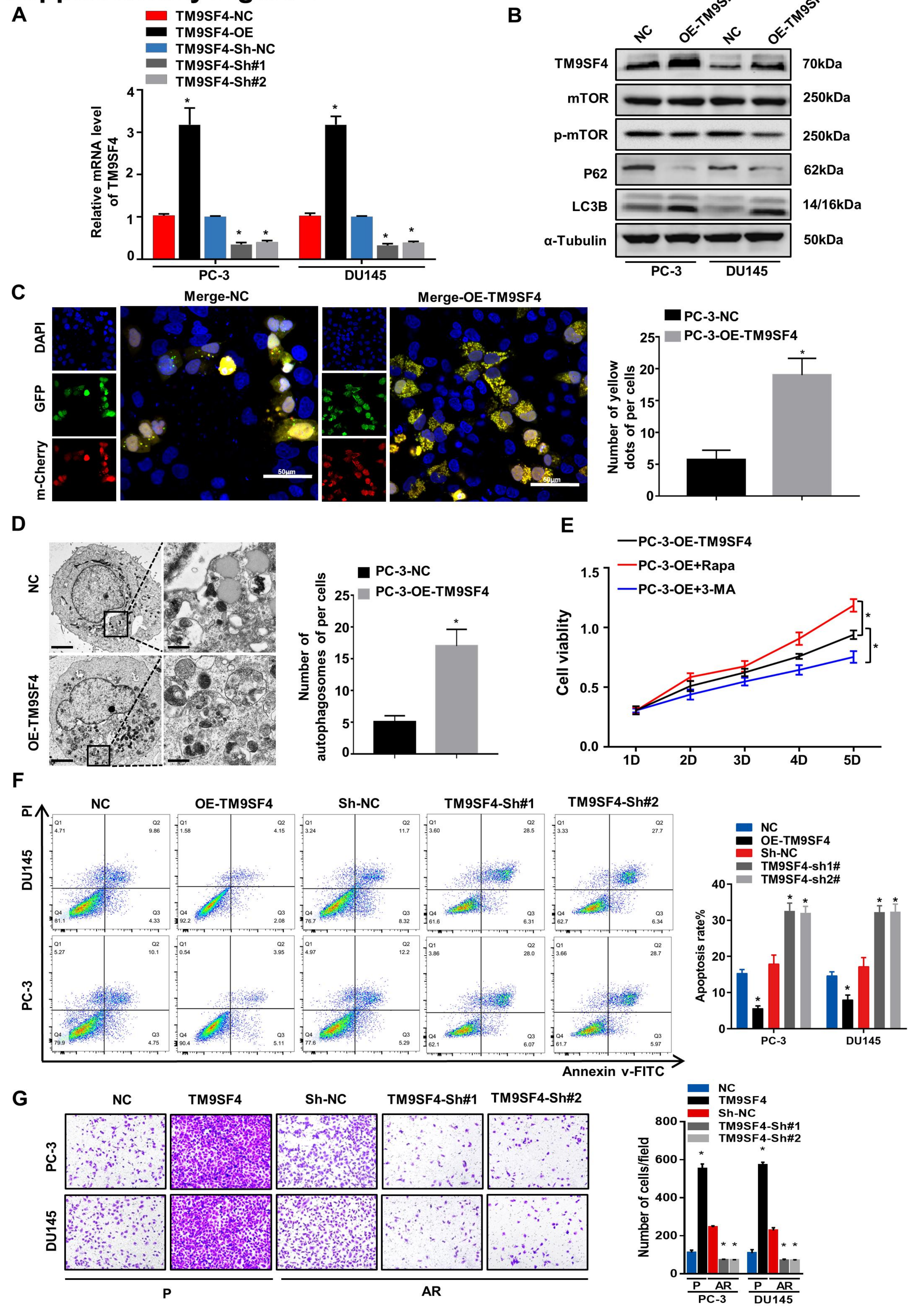

Supplementary Figure 1: Anoikis-resistant PCa cells enhance the capabilities of survival and migration.

(A) Representative pictures (left panel) and percentage of the original wound area (right panel) of parental (P) and anikis-resistant (AR) PCa cells (PC-3、DU145) analyzed using a wound-healing assay (Scale bar, 100 μm).

(B) Representative pictures (left panel) and quantification (right panel) of migratory parental (P) and anikis-resistant (AR) PCa cells (PC-3、DU145) analyzed using a transwell migration assay (Scale bar, 100 μm).

(C) The protein levels of CEMIP, MMP2, and MMP9 detected by western-blotting in anikis-resistant (P) and parental (AR) PCa cells.

(D) Apoptosis was assessed by flow cytometry assay in anikis-resistant and parental PCa cells. Bar graphs show the statistical analysis of three independent experiments (* $P < 0.05$).

Supplementary Figure 2: Circ_0004585 promotes PCa cells migration, invasion, anikis-resistance.

(A) Representative images (left panel) and quantification (right panel) results of flow cytometry assay for the apoptosis rates of PC-3 and DU145 cells upon circ_0004585 overexpression or knockdown.

(B) Representative pictures and percentage of the original wound area of PC-3 cells with circ_0004585 overexpression or knockdown analyzed using a wound-healing assay (Scale bar, 100 μm).

(C) Representative images results of transwell migration assays for PC-3 and DU145 cells with circ_0004585 overexpression or knockdown (Scale bar, 100 μm). Bar graphs show the statistical analysis of three independent experiments (* $P < 0.05$).

(D) Western blot was performed to evaluate the expression of LC3II/I, MMP2 and MMP9 in overexpressed circ_0004585 PC-3 cells with autophagy activator rapamycin or autophagy inhibitor 3-MA for 24 h.

(E) Flow cytometry was used to detect the apoptosis level of PCa cells with stable overexpression of circ_0004585 after the addition of rapamycin or CQ for 24 h, * $P < 0.05$; ** $P < 0.01$.

(F) Transwell assay was used to detect the migration cells of PCa cells with stable overexpression of circ_0004585 after the addition of CQ for 24 h, (* $P < 0.05$; ** $P < 0.01$).

Supplementary Figure 3: Upregulation of TM9SF4 promotes the invasion, migration and anoikis-resistance of prostate cancer cells by activating autophagy

(A) qRT-PCR was performed to evaluate the expression of TM9SF4 in PC-3 and DU145 cells which were transfected with the indicated plasmids. GAPDH was used as internal control.

(B) Western blot was performed to evaluate the expression of TM9SF4, mTOR, p-mTOR, P62, and LC3BII/I in PC-3 and DU145 cells which were transfected with the indicated plasmids.

(C) Autophagic flux was monitored in stable up-regulated TM9SF4 PC-3 cells expressing endogenous LC3B tagged with tandem fluorescent-mCherry-GFP as a reporter, GFP and mCherry signal colocalization (yellow dots) indicated the lack of phagophore or autophagosome fusion with lysosomes (Scale bar, 50 μm).

(D) Transmission electron microscopy (TEM) revealed the number of double-membrane autophagosomes in stable overexpressed TM9SF4 PC-3 cells (Original

magnification, ×1000, ×1600, respectively).

(E) The cell viability rate was detected using CCK-8 after individually adding rapa or 3-MA to the PCa cells 24 h displaying stable TM9SF4 overexpression.

(F) Representative images results of flow cytometry assay for the apoptosis rates of PC-3 and DU145 cells upon TM9SF4 overexpression or knockdown.

(G) Representative pictures of PC-3 cells with circ_0004585 overexpression or knockdown analyzed using a transwell migration assay (Scale bar, 100 μm). The results were derived from three independent experiments. Data are presented as the means ± SEM of three independent experiments. *P < 0.05; **P < 0.01 (Student's t-test).

Supplementary Table 1: qRT-PCR primer sequences in this study

Primer names	Primer sequence
GAPDH-F	GGTCGGAGTCAACGGATTTG
GAPDH-R	GGAAGATGGTGATGGGATTTC
Circ_0004585-Con-F	AAGGAGGCGCTCTTGAGTTG
Circ_0004585-Con-R	ACTGTGCCTGATTTGGGGTC
Circ_0004585-Di-F	TGGCCTCCTTGTCAAGTCTG
Circ_0004585-Di-R	GGGAAGCAGGTCAGAGTGAG
Circ_0002970-F	TACCCGATTCACTTCCACCTG
Circ_0002970-R	TAGCATTTGTCCTCCATCTCCC
Circ_0003893-F	AGCCACTACTACTGGGACGA
Circ_0003893-R	GTAATGGGTGCTCCTGGTGA
MiR-1248-F	GCGACCTTCTTGTATAAGCACTGT
MiR-1248-R	AGTGCAGGGTCCGAGGTATT
MiR-1231-F	GCGGTGTCTGGGCGGAC
MiR-1231-R	AGTGCAGGGTCCGAGGTATT
MiR-338-3p-F	CGCGTCCAGCATCAGTGATT
MiR-338-3p-R	AGTGCAGGGTCCGAGGTATT
MiR-657-F	CGGGCAGGTTCTCACCCTC
MiR-657-R	AGTGCAGGGTCCGAGGTATT

MiR-1265-F	GCGCAGGATGTGGTCAAGT
MiR-1265-R	AGTGCAGGGTCCGAGGTATT
MiR-661-F	TGCCTGGGTCTCTGGCCT
MiR-661-R	AGTGCAGGGTCCGAGGTATT
MiR-339-3p-F	TGAGCGCCTCGACGACA
MiR-339-3p-R	AGTGCAGGGTCCGAGGTATT
MiR-1182-F	GGAGGGTCTTGGGAGGGA
MiR-1182-R	AGTGCAGGGTCCGAGGTATT
MiR-663b-F	GGTGGCCCGGCCGTGC
MiR-663b-R	AGTGCAGGGTCCGAGGTATT
MiR-648-F	CGCGAAGTGTGCAGGGC
MiR-648-R	AGTGCAGGGTCCGAGGTATT
MiR-615-5p-F	GGGGGTCCCCGGTGCT
MiR-615-5p-R	AGTGCAGGGTCCGAGGTATT
MiR-1289-F	GCGTGGAGTCCAGGAATCTG
MiR-1289-R	AGTGCAGGGTCCGAGGTATT
TM9SF4-F	GATTGGTTGCCGTGGTCTTTA
TM9SF4-R	TTCTACGGGATCGTTCTGGTG
CEMIP-F	GGCCGGTGATGTAGACGAAA
CEMIP-R	CCATTGGAGCCATGGACTGT

Supplementary Table 2: Drugs and reagents

Drug / Reagent	Source	Identifier / formulation
FITC-Annexin V apoptosis	BD Biosciences kit	Catalog No: 556547
PE-Annexin V apoptosis detection	BD Biosciences kit	Catalog No: 559763
CCK-8	Vazyme Biotech	Catalog No: A311-01
rapamycin	Selleck	Catalog No: S1039
3-Methyladenine	Selleck	Catalog No: S2767

Supplementary Table 3：Primary and secondary antibodies

Antibody	Source	Identifier	Host
CEMIP antibody	Abcam	Catalog No: ab62322	Rabbit
TM9SF4 antibody	Proteintech	Catalog No: 25595-1-AP	Rabbit
mTOR antibody	Proteintech	Catalog No: 66888-1-Ig	Rabbit
p-mTOR antibody	CST	Catalog No: 5536S	Mouse
S6K1 antibody	Proteintech	Catalog No: 14485-1-AP	Rabbit
p-S6K1 antibody	CST	Catalog No: 9204S	Rabbit
4E-BP1 antibody	CST	Catalog No: 9644S	Rabbit
p-4E-BP1 antibody	CST	Catalog No: 2855S	Rabbit
P62 antibody	Proteintech	Catalog No: 18420-1-AP	Rabbit
LC3B antibody	Abcam	Catalog No: ab192890	Rabbit
α-Tubulin antibody	Proteintech	Catalog No: 66031-1-Ig	Mouse

488 - Anti-Mouse IgG(H+L)	Proteintech	Catalog No: SA00013-1	Mouse
594 –Anti-Rabbit IgG(H+L)	Proteintech	Catalog No: SA00013-4	Rabbit

Supplementary Table 4：Probe sequence in this study

Probe name	probe sequence
Circ_0004585	5’ TCCTGGCAGTGTGCTCCTTGCAGTCTTGCCTGGG-biotin
oligo probe	5’ CCCAGGCAAGACTGCAAGGAGCACACTGCCAGGA-biotin

Supplementary Table 5：TM9SF4 shRNA sequence in this study

TM9SF4	shRNA sequence
ShRNA1	5′-GCGGATCACAGAAGACTACTA-3′
ShRNA2	5′-CGGTGGTACATGAACCGATTT-3′.
